# Novel Clinical Trial Designs for Intermediate Age-Related Macular Degeneration

**DOI:** 10.1016/j.xops.2026.101179

**Published:** 2026-03-30

**Authors:** Alessandro Berni, Mengxi Shen, Jeremy Liu, James D. Kastner, Omar S. El-Mulki, Sara Beqiri, Gissel Herrera, Omer Trivizki, Nadia K. Waheed, Dale W. Usner, Brian Levy, Robert O’Brien, Giovanni Gregori, Philip J. Rosenfeld

**Affiliations:** 1 Department of Ophthalmology, Bascom Palmer Eye Institute, University of Miami Miller School of Medicine, Miami, Florida; 2 Department of Ophthalmology, IRCCS San Raffaele Scientific Institute, Milan, Italy; 3 Department of Ophthalmology and Visual Science, Yale University School of Medicine, New Haven, Connecticut; 4 Department of Ophthalmology, Tel Aviv Medical Center, University of Tel Aviv, Tel Aviv, Israel; 5 New England Eye Center, Tufts Medical Center, Tufts University School of Medicine, Boston, Massachusetts; 6 Ophthalstat, Athol, Idaho; 7 InflammX Therapeutics, Inc, Tampa, Florida

**Keywords:** Age-related macular degeneration, Clinical trials, OCTA, En face imaging

## Abstract

**Purpose:**

To develop and model clinical trial designs for intermediate age-related macular degeneration (iAMD) using OCT-based structural biomarkers to define inclusion criteria and estimate sample size requirements for detecting treatment effects over 2 years.

**Design:**

Retrospective study of a prospectively acquired swept-source OCT cohort.

**Subjects:**

Patients with iAMD enrolled in a prospective natural history imaging study.

**Methods:**

Two clinical trial designs were modeled. The first included eyes with drusen volume (DV) ≥0.20 mm^3^ and any hyperreflective foci (HRF). The second included eyes with an area of HRF ≥0.07 mm^2^, regardless of DV. Eyes were followed from the time of eligibility and monitored for the onset and growth of large hypertransmission defects (hyperTDs). Power and sample size simulations were performed based on the expected treatment effects.

**Main Outcome Measures:**

Annual square root growth rates of large hyperTDs and incidence of new large hyperTDs over 2 years.

**Results:**

In 76 eyes with DV ≥0.20 mm^3^ and HRF, the mean annual growth rate of large hyperTDs was 0.153 mm/year (standard deviation [SD] = 0.188), and 35.5% of these iAMD eyes developed hyperTDs by 2 years. In the 79 eyes with HRF area ≥0.07 mm^2^, the mean annual growth rate was 0.161 mm/year (SD = 0.172), and 40.5% developed hyperTD by 2 years. With a 2-sided alpha of 0.1, a 50% reduction in the growth rate could be detected with 80% power using ≥89 eyes per arm in the first group and ≥64 eyes per arm in the second group.

**Conclusions:**

The growth rate of large hyperTDs is a continuous and reproducible structural endpoint for iAMD trials, influenced by both lesion onset and progression. Drusen volume or the area of HRF serves as high-risk biomarkers for enrolling iAMD eyes. This design allows for a study duration of 1–2 years with no more than 100 subjects per arm.

**Financial Disclosure(s):**

Proprietary or commercial disclosure may be found in the Footnotes and Disclosures at the end of this article.

Age-related macular degeneration (AMD) is the leading cause of irreversible legal blindness among the elderly worldwide.^1^ Legal blindness occurs during the late stages of AMD and is characterized by the onset and progression of geographic atrophy (GA) or macular neovascularization.^2^ Both GA and macular neovascularization arise from intermediate AMD (iAMD).^2^ While inhibitors of VEGF (anti-VEGF therapy) effectively suppress exudation in neovascular AMD, they do not alter the underlying progression of the disease to GA or prevent the enlargement of GA present at the time of exudation.^3^ Although complement inhibition slows the enlargement of GA, this treatment does not stop disease progression or restore vision already lost from AMD.^4^, ^5^, ^6^ For these reasons, there is a need to treat AMD at an earlier stage and identify patients at high risk for disease progression from iAMD to late AMD so that they can be enrolled in interventional studies.

OCT imaging has become the gold standard for identifying and monitoring anatomic changes that occur as AMD progresses.^7^^,^^8^ Several OCT biomarkers have been proposed as useful predictors of disease progression from iAMD to GA.^9^^,^^10^ These biomarkers include the total burden of macular drusen,^11^, ^12^, ^13^ the macular area of hyperreflective foci (HRF),^14^^,^^15^ the presence of calcified or hyporeflective-core drusen,^16^, ^17^, ^18^, ^19^ vitelliform lesions,^20^ reticular pseudodrusen, also known as subretinal drusenoid deposits,^21^ and the decrease in macular choriocapillaris perfusion.^22^, ^23^, ^24^, ^25^

The total burden of central macular drusen is a defining feature of iAMD and a well-established risk factor for disease progression.^11^, ^12^, ^13^ Prior natural history studies using swept-source OCT (SS-OCT) have shown that a high drusen volume (DV) identifies eyes at increased risk for the onset and progression of large choroidal hypertransmission defects (hyperTDs), which represent an early structural precursor of GA.^13^^,^^26^^,^^27^ Subsequent work demonstrated that the HRF area represents a complementary OCT biomarker, identifying a subset of eyes that progress to hyperTDs even in the absence of a large drusen burden.^14^ Together, these findings support the concept that OCT-based risk enrichment can substantially increase the probability of observing meaningful structural progression within a clinically feasible timeframe.

Discussions at the 2024 Ryan Initiative for Macular Research meeting highlighted the need for clinical trial designs that efficiently enrich for iAMD eyes likely to progress within 1–2 years.^28^ Based on this consensus, we propose 2 OCT-based phase II trial designs targeting high-risk iAMD populations. The first design builds on a DV-based enrichment strategy (central DV ≥0.20 mm^3^) by additionally requiring the presence of HRF at baseline. The second design selects eyes based on a predefined HRF area threshold, independent of DV. In both designs, the primary efficacy endpoint is the growth rate of large hyperTDs over 2 years, with hyperTD incidence at 1 and 2 years as secondary endpoints. This endpoint integrates both lesion onset and subsequent enlargement into a single continuous progression metric, enabling detection of treatment effects over shorter follow-up durations. Interim analyses of hyperTD incidence may further inform subsequent phase III trial planning.

Our natural history study of eyes with iAMD allowed us to identify high-risk OCT biomarkers in iAMD eyes that predicted disease progression. The models that we developed based on these OCT biomarkers enabled us to simulate different rates for the appearance and growth of large hyperTDs. Applying our natural history data and simulations using defined power and significance levels, we estimated the number of subjects needed in each treatment arm of the proposed clinical trials.

## Methods

This work has 2 components. First, we analyzed a prospectively acquired SS-OCT natural history cohort to quantify DV, HRF area, and the onset and longitudinal enlargement of large hyperTDs, and to estimate per-eye progression slopes. Second, we used these empiric natural history estimates to parameterize 2 proposed phase II trial designs, including endpoint definition, analytical framework for a future interventional trial, and sample size/power calculations.

### Participants

A prospective SS-OCT imaging study was approved by the ethics committee of the University of Miami Miller School of Medicine. All participants provided informed consent, and the study was performed following the tenets of the Declaration of Helsinki and complied with the Health Insurance Portability and Accountability Act of 1996. The study involved a retrospective review of a prospectively enrolled SS-OCT imaging database at the Bascom Palmer Eye Institute, including patient data collected between April 2016 and June 2023. To be included in the study, patients were required to have a diagnosis of iAMD in ≥1 eye and a minimum of 1 year of follow-up from the baseline visit to the latest assessment. Intermediate AMD was defined by the presence of ≥1 large drusen with a minimum diameter of 125 μm within the central foveal-centered 5-mm circle on the OCT scan. The diagnosis of iAMD and confirmation of eligibility criteria were performed by 2 experienced graders (J.L. and M.S.) working by consensus on OCT imaging, with adjudication by a senior grader (P.J.R.) when required. No interventional treatments for iAMD were administered to the study eye before or during the follow-up period. The use of nutritional supplements (e.g., the Age-Related Eye Disease Study formulation) would be expected in this population of patients with high-risk iAMD. While we currently have no evidence that nutritional supplements slow the formation of large hyperTDs, future studies could stratify patients at baseline according to whether they consumed nutritional supplements before or during the follow-up period. The included eye could not have a history of exudative AMD, or the presence of GA, defined as a well-demarcated area of retinal pigment epithelium (RPE) loss/attenuation resulting in a choroidal hyperTD with a greatest linear dimension (GLD) ≥250 μm on en face images, which has been shown to be consistent with early or more advanced GA.^13^^,^^26^^,^^27^ Patients with diabetic retinopathy, pathological myopia, or other retinal conditions associated with drusen-like deposits, such as Stargardt disease or vitelliform dystrophy, were excluded. Additionally, patients with significant vitreoretinal interface abnormalities that distorted the macular anatomy or those who had previously undergone vitreoretinal surgery were also excluded.

### Imaging

All eyes included in the study underwent SS-OCT imaging at each follow-up visit using the PLEX Elite 9000 instrument (Carl Zeiss Meditec). Imaging was performed by one of 3 trained technicians. The SS-OCT system utilized a swept-source laser with a central wavelength of 1050 nm and a bandwidth of 100 nm. The device provided a resolution of approximately 5 μm axially and 20 μm transversally at the retinal surface. Imaging was conducted using the SS-OCT angiography (SS-OCTA) 6 × 6 mm scan pattern, centered on the fovea, with a scanning speed of 100 000 A-scans per second. The protocol involved 500 A-scans per B-scan, with each B-scan being repeated twice at the same location for angiographic signal acquisition, and 500 B-scan positions along the perpendicular slow axis, maintaining a uniform 12 μm spacing between A-scans. Each A-scan comprised 1536 pixels over a depth of 3 mm. All volumetric data sets were evaluated for quality and signal strength, and any scans with a signal strength <7 or significant motion artifacts were excluded from analysis. In cases where multiple scans of the same type were available, the highest-quality scan was selected for analysis. The Advanced RPE Analysis algorithm version 0.10 (Carl Zeiss Meditec) was applied to all scans to calculate the DV within a 5-mm circle centered on the fovea at each visit, following the methodology described by Jiang et al.^29^ All scans were examined for nonexudative macular neovascularization using 2 specific SS-OCTA en face slabs: the outer retina–choriocapillaris slab and a second slab spanning from the RPE to Bruch’s membrane.^30^^,^^31^ All images were carefully reviewed for any signs of exudation, defined as the presence of subretinal or intraretinal fluid visible on structural OCT B-scans and retinal thickness maps.

### Grading of Large Choroidal hyperTDs

Large choroidal hyperTDs were defined as areas of increased focal brightness, reflecting enhanced light transmission into the choroid with a GLD of ≥250 μm as outlined in previous studies.^26^^,^^27^^,^^32^ These large hyperTDs were detected using en face structural images derived from a sub-RPE slab positioned 64 μm to 400 μm beneath Bruch’s membrane. Every visit for each eye was reviewed by 2 of 3 independent graders (A.B., J.L., or M.S.) to identify the first and any additional large hyperTDs. The GLD of each lesion was measured using the caliper tool provided by the imaging device’s software. Large hyperTDs were delineated on the sub-RPE slabs, and corresponding B-scans were examined to confirm their presence. The graders reached consensus on the manual delineation of each lesion, with any disagreements resolved by a senior grader (P.J.R.). Each identified large hyperTD was given a unique identifier and tracked over time to monitor persistence, growth, and potential merging into larger choroidal hyperTDs.

### HRF Area Measurements

The presence of HRF prevents OCT signal penetration into the choroid, resulting in choroidal hypotransmission defects (hypoTDs) on en face sub-RPE slabs, which appear as dark areas compared to the surrounding tissue.^33^ Using a validated semiautomated algorithm, as previously described, we identified and outlined hypoTDs attributable to HRF at each follow-up visit.^14^^,^^34^ Because hypoTDs in nonexudative AMD eyes can also be caused by extensive pigment epithelial detachments, vitelliform lesions, and calcified drusen, corresponding B-scans were consistently reviewed for confirmation. Hyperreflective foci included both HRF located along the RPE, which appear on structural OCT B-scans as focal areas of increased RPE hyperreflectivity or thickness, and intraretinal HRF, which manifest as well-defined focal lesions within the neurosensory retina with reflectivity equal to or greater than that of the RPE.^14^^,^^33^ The algorithm employed depth-resolved segmentation based on the optical attenuation coefficient to detect hyperreflective regions corresponding to these lesions.^34^ After matching HRF with their corresponding hypoTDs, the algorithm generated initial outlines of these HRF areas on the sub-RPE slabs. Only hypoTDs covering an area of ≥1440 μm^2^, corresponding to ≥10 pixels on the 6 × 6 mm SS-OCTA images, were included, as this was the smallest area reproducibly measurable by graders. Two independent graders (A.B. and M.S.) manually reviewed all algorithm-generated outlines using en face and B-scan images, making necessary corrections with a built-in editing tool. In cases where consensus between the graders could not be reached, a senior evaluator (P.J.R.) adjudicated the discrepancies. Finally, the area measurements (mm^2^) of HRF regions within a 5-mm fovea-centered circle were recorded at each visit.

All quantitative analyses were restricted to a 5-mm fovea-centered circle. This region was selected because it represents the largest area that can be reliably and consistently analyzed within standard 6 × 6 mm OCT scans across longitudinal follow-up, while maintaining biological relevance, complete spatial coverage, and consistency with prior studies.^8^^,^^11^^,^^14^^,^^15^^,^^19^^,^^23^^,^^24^^,^^35^, ^36^, ^37^, ^38^, ^39^, ^40^

### Modeling Clinical Trials in iAMD

To inform trial design, we defined 2 high-risk eligibility profiles within the natural history cohort and used these inclusion criteria as the analytic baseline. All progression estimates reported here were derived from the natural history data; these empiric estimates were subsequently used as inputs for trial simulations and sample size calculations. Because participants in the natural history imaging cohort could contribute one or both eligible eyes, analyses were initially performed at the eye level. To assess the impact of within-person correlation when both eyes were included, linear mixed-effects models with subject-level random intercepts and slopes were also fitted. These models yielded estimates of progression variability that were very similar to eye-level analyses, indicating minimal impact of within-subject correlation on progression estimates. In the proposed clinical trial designs, only one study eye per participant would be enrolled, and all sample size and power calculations are therefore reported as eyes per arm under the assumption of enrolling a single study eye per participant.

### Data Analysis of the Natural History Cohort

For both eligibility criteria, eyes with any large hyperTDs present at baseline were excluded, and only eyes without large hyperTDs at baseline were eligible for modeling. The first eligibility profile included iAMD eyes with a DV of ≥0.20 mm^3^ and any detectable HRF (HRF area >0 mm^2^), both measured within the 5-mm circle centered on the fovea on the same 6 × 6 mm OCT raster scan without any concurrent large hyperTD at baseline. The second eligibility profile included eyes with no large hyperTDs at baseline and an area of HRF ≥0.07 mm^2^ within the same 5-mm circle, independent of DV. For both designs, the modeling was based on the natural history of eyes in our prospective SS-OCT imaging study, meeting these criteria at the time of their inclusion into the natural history study or at some point during their follow-up. The first visit at which inclusion criteria were met was designated as the analytic baseline for that eligibility profile. Only eyes having ≥1 year of follow-up from the analytic baseline to their last visit were included. The frequency of evaluations for patients included in the study reflected routine clinical care and was influenced by disease progression in the study eye, the condition of the fellow eye, and the patient's availability for follow-up visits. As a result, follow-up intervals varied among patients.

Progression of large hyperTDs was assessed using the square root of the total area of the large hyperTDs within an eye, regardless of the number of large hyperTDs contributing to that total value. The square root transformation is known to reduce the dependency of the growth rate on the baseline size.^41^^,^^42^

We calculated the annual rate of change of the square root total area of the large hyperTDs in each eye using a least-squares regression (LSR) approach. This slope-based approach allowed us to capture continuous progression over time, regardless of whether a subject developed large hyperTDs.

For each eye, an LSR model was fitted to the square root total area measurements of the large hyperTDs at all available time points, from the baseline (inclusion) visit through their last available follow-up. Importantly, all follow-up visits were included, even if the large hyperTD area was zero. This ensured that subjects who did not develop large hyperTDs during follow-up were still incorporated into the modeling with a slope of 0, which is critical for preserving the proper distribution of progression rates in the population. The resulting individual regression slopes (mm/year) were summarized empirically (mean/standard deviation [SD] and percentiles) and then used to model the probabilistic distribution of measurements for the simulated samples generated to estimate the power of the proposed clinical trials, given an expected treatment effect. The probabilistic distributions of the LSR slopes were based on natural history data in eyes with ≥1 year of follow-up and are expected to produce a good estimate of possible study outcomes.

To accommodate irregular follow-up intervals and summarize average natural history progression, we additionally fitted a linear mixed-effects model with both a random intercept and a random slope for time, using the square root–transformed total large hyperTD area as the continuous outcome. This model was applied as a secondary sensitivity analysis to provide population-averaged estimates of progression while accounting for within-eye correlation and variable visit schedules. Importantly, the mixed-effects model assumes a continuous Gaussian distribution of subject-specific slopes and therefore does not preserve a point mass at zero for eyes that do not develop large hyperTDs during follow-up. For this reason, the LSR-derived per-eye slopes, which explicitly include all visits with area = 0 and allow nondeveloping eyes to contribute a slope of exactly zero, define the primary estimand used for trial simulation and design. Two inclusion definitions were considered: (1) eyes with DV ≥0.20 mm^3^ and any HRF >0 mm^2^ and (2) eyes with HRF area ≥0.07 mm^2^. For each definition, the mixed-effects model estimated the mean annual change in square root of hyperTD area across subjects. This approach was applied as a sensitivity analysis for the natural history data to provide population-averaged estimates while accommodating irregular visit schedules. In future interventional trials, the primary analysis model would be prespecified in the protocol; in this design work, simulations were based on the LSR-derived per-eye slope estimand.

### Clinical Trial Design and Sample Size Modeling

Using the empiric natural history slope estimates from the aforementioned LSR analyses, we calculated sample sizes under prespecified treatment effects and statistical assumptions.

By using SAS Proc Power software and estimates of the mean and SD of the annual slope of the square root large hyperTD area progression, we calculated the required sample sizes under the following assumptions: (1) 25%, 30%, 35%, 40%, 45%, and 50% reduction in the annual rate of change in the treatment arm compared with placebo; (2) a 2-sided alpha level of 0.15, 0.10, or 0.05; and (3) 80% power. To evaluate the potential impact of variability across study centers, additional scenarios were modeled assuming 10%, 20%, and 30% increases in the SD of the annual slope estimates. The required sample sizes were calculated under the specified assumptions, as well as with a 10% inflation factor to account for potential attrition or protocol deviations. The assumed true difference in mean progression and the assumed common SD were used to determine sample size, assuming a t-distribution around the mean based on the central limit theorem. These calculations target differences in slope rather than fixed time-point changes and remain valid across varying trial durations, provided that sufficient longitudinal data are available to reliably estimate individual slopes. Sample size planning was primarily based on simulations derived from empirical LSR slope distributions, which include a spike at zero reflecting delayed lesion onset, while *t* test–based calculations are presented as approximate summaries for interpretability.

## Results

### Study Population

A total of 171 eyes from 121 patients were included in a retrospective analysis of a prospectively enrolled natural history study of eyes with iAMD. At baseline, the mean age of participants was 74.0 ± 7.3 years, and women constituted 64% of the data set. The median duration of follow-up until the first large hyperTD or last visit was 59.1 months (95% confidence interval [CI]: 52.0–67.8 months). OCT images were acquired at various follow-up intervals in accordance with the patients' clinical management. Overall, patients had a mean of 14.4 ± 8.4 visits during their entire follow-up period, with a mean of 3.3 ± 1.4 visits per year. During the follow-up period, 19 eyes (11.1%) from 17 patients developed exudative AMD and were subsequently excluded from further examination.

### Trial Design 1: DV ≥0.20 mm^3^ and HRF >0 mm^2^

**Figure 1:**
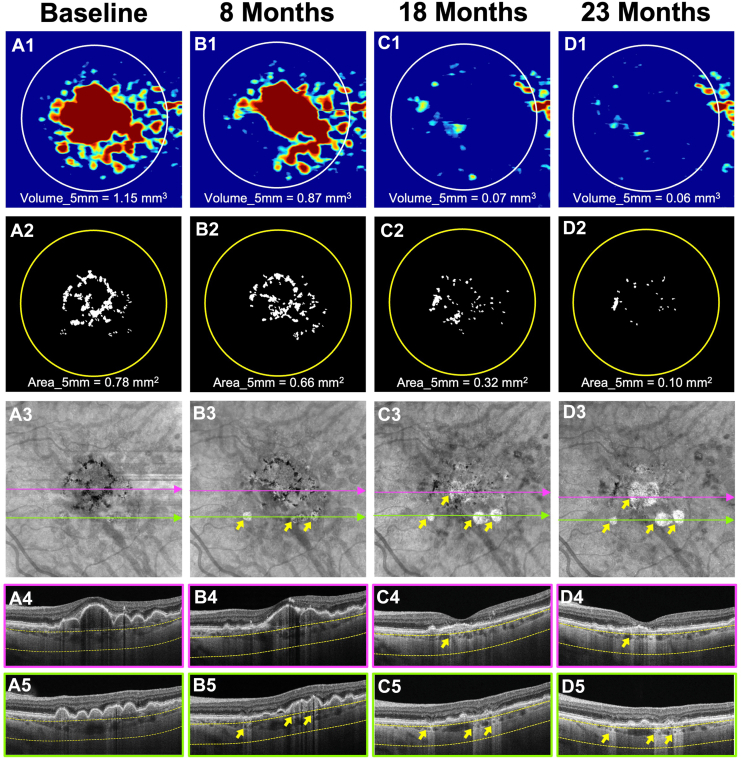
Longitudinal SS-OCTA imaging from an iAMD eye with a baseline drusen volume ≥0.20 mm^3^ and HRF area >0 mm^2^. Column **A** (A1–A5) represents the baseline visit. Column **B** (B1–B5) represents the 8-month follow-up visit. Column **C** (C1–C5) represents the 18-month follow-up visit. Column **D** (D1–D5) represents the 23-month follow-up visit. Row 1 (A1, B1, C1, D1) shows the drusen volume maps with volume quantification in the 5-mm fovea-centered circle. Row 2 (A2, B2, C2, D2) shows the final outlines with HRF area measurements in the 5-mm circle centered on the fovea. Row 3 (A3, B3, C3, D3) shows the en face SS-OCTA sub-RPE slabs obtained using segmentation boundaries between 64 and 400 μm under Bruch’s membrane. Choroidal hypotransmission defects caused by HRF can be seen on en face sub-RPE slabs and corresponding color-framed B-scans (rows 4–5). At baseline (A1–A5), the eye showed large drusen (A1) with an extensive HRF area (A2). After 8 months of follow-up (B1–B5), both drusen volume (B1) and HRF area (B2) decreased, while 3 large hyperTDs emerged (yellow arrows, B3 and B5). By the 18-month follow-up, drusen were nearly undetectable (C1), and the HRF area continued to decrease (C2). Additional large hyperTDs formed, and the previously developed large hyperTDs enlarged (yellow arrows, C3). At the 23-month visit, all the large hyperTDs had enlarged further (yellow arrows, D3), alongside continued reductions in drusen volume (D1) and HRF area (D2). HRF = hyperreflective foci; hyperTD = hypertransmission defect; iAMD = intermediate age-related macular degeneration; RPE = retinal pigment epithelium; SS-OCTA = swept-source OCT angiography.

**Figure 2:**
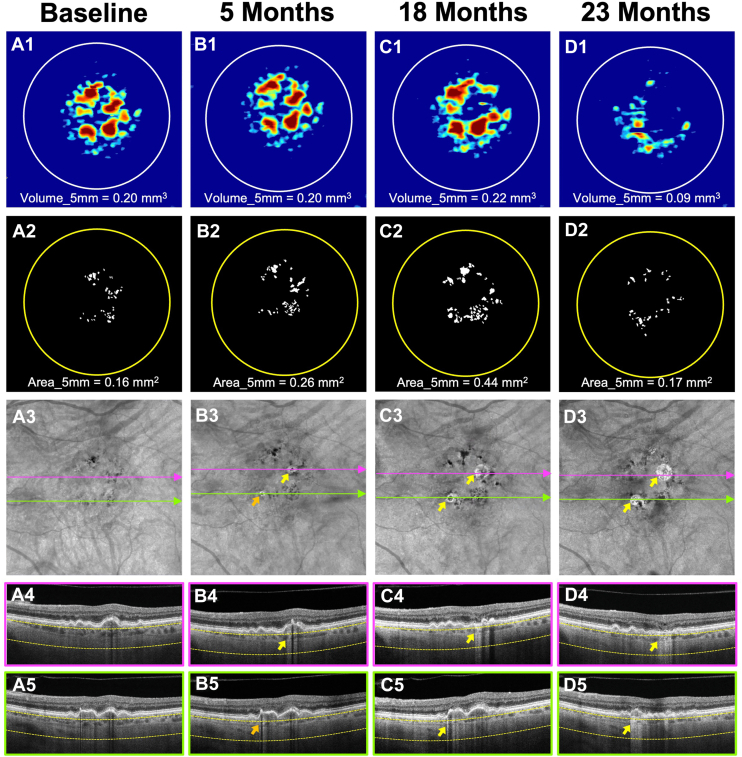
Longitudinal SS-OCTA imaging from an iAMD eye meeting the baseline drusen volume (≥0.20 mm^3^) and HRF area (>0 mm^2^) criteria for enrollment. Column **A** (A1–A5) represents the baseline visit. Column **B** (B1–B5) represents the 5-month follow-up visit. Column **C** (C1–C5) represents the 18-month follow-up visit. Column **D** (D1–D5) represents the 23-month follow-up visit. Row 1 (A1, B1, C1, D1) shows the drusen volume maps with volume quantification in the 5-mm fovea-centered circle. Row 2 (A2, B2, C2, D2) shows the final outlines with HRF area measurements in the 5-mm circle centered on the fovea. Row 3 (A3, B3, C3, D3) shows the en face SS-OCTA sub-RPE slabs obtained using segmentation boundaries between 64 and 400 μm under Bruch’s membrane. Choroidal hypotransmission defects caused by HRF can be seen on en face sub-RPE slabs and corresponding color-framed B-scans (rows 4–5). At baseline (A1–A5), the eye showed a drusen volume of 0.20 mm^3^ (A1) with an HRF area of 0.16 mm^2^ (A2) within the 5-mm circle. After 5 months of follow-up (B1–B5), the drusen volume remained stable (B1), while the HRF area (B2) increased. A large hyperTD emerged (yellow arrow, B3), and another began forming but had not yet reached 250 μm in GLD (orange arrow). By the 18-month follow-up, both drusen volume and HRF area had further increased (C1 and C2), and the previously developed large hyperTDs continued to enlarge (yellow arrows, C3). At the 23-month visit, both large hyperTDs showed continued growth (yellow arrows, D3), while drusen volume (D1) and HRF area (D2) decreased. GLD = greatest linear dimension; HRF = hyperreflective foci; hyperTD = hypertransmission defect; iAMD = intermediate age-related macular degeneration; RPE = retinal pigment epithelium; SS-OCTA = swept-source OCT angiography.

A total of 76 eyes from 60 patients met the eligibility criteria of having a DV ≥0.20 mm^3^ and an HRF area >0 mm^2^, either at baseline or during follow-up, but prior to the onset of their first large hyperTD. Of these patients, 63.1% were women. The median follow-up time to last visit was 37.7 months (95% CI: 32.2–50.1 months). By 12 months, 15 eyes (19.7%) had developed a large hyperTD, increasing to 27 eyes (35.5%) by 24 months. Forty-six (60.5%) of the 76 eyes developed ≥1 large hyperTD during follow-up and had nonzero LSR slope values. The median time to a large hyperTD onset was 20.4 months (95% CI: 12.3–27.4 months). The estimated mean annual slope of the square root–transformed large hyperTD area progression among all 76 eyes was 0.153 mm/year (SD = 0.188 at the eye level). To simulate potential treatment effects, we modeled reductions in the annual rate of change starting from 25%, corresponding to a mean difference of 0.0375 mm/year, to a 50% reduction, corresponding to a mean difference of 0.0750 mm/year, between treatment and placebo arms (Table 1). Power calculations were then performed using these estimates in combination with a range of assumed SDs (0.18, 0.19, and 0.20) to reflect variability that could reasonably be observed in a multicenter clinical trial setting. This range was chosen to account for potential differences in measurement consistency, imaging conditions, and population characteristics relative to the modeling data set. Figures 1 and 2 illustrate representative eyes with baseline DV ≥0.20 mm^3^ and HRF area >0 mm^2^ that developed large hyperTDs within 2 years of enrollment.

**Table 1:** Estimated Treatment Effects and Study Population Characteristics for the Proposed iAMD Clinical Trial Designs

Trial Design	Total Number of Eyes; Eyes Developing hyperTDs	Median Follow-Up; months (95% CI)	Median Number of Visits per Eye (95% CI)	Slope Mean (SD); mm/Year	Reduction in Annual Rate of Change Active Arm vs. Control; mm/Year					
					25%	30%	35%	40%	45%	50%
DV ≥0.20 mm^3^; HRF area >0 mm^2^	76; 46 (60.5%)	37.7 (32.2–50.1)	11.5 (9.5–15)	0.153 (0.188)	0.0375	0.0450	0.0525	0.0600	0.0675	0.0750
HRF area ≥0.07 mm^2^	79; 53 (67.1%)	35.41 (31.8–47.4)	12 (8–14)	0.161 (0.172)	0.0400	0.0480	0.0560	0.0640	0.0720	0.0800

CI = confidence interval; DV = drusen volume; HRF = hyperreflective foci; hyperTD = hypertransmission defect; iAMD = intermediate age-related macular degeneration; SD = standard deviation.

### Trial Design 2: HRF Area ≥0.07 mm^2^

**Figure 3:**
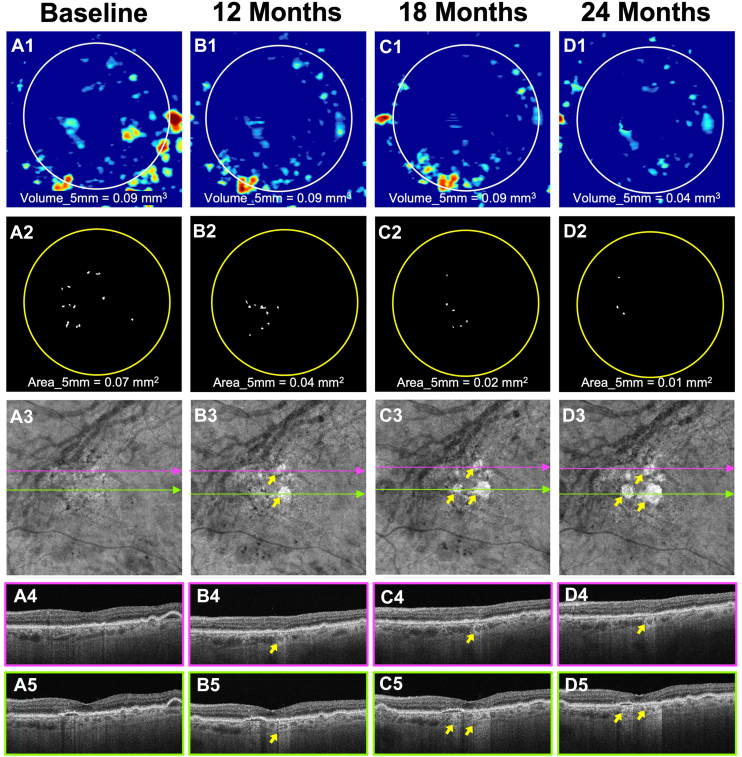
Longitudinal SS-OCTA imaging from an iAMD eye meeting the baseline HRF area (≥0.07 mm^2^) criterion for enrollment in the second clinical trial design. Column **A** (A1–A5) represents the baseline visit. Column **B** (B1–B5) represents the 12-month follow-up visit. Column **C** (C1–C5) represents the 18-month follow-up visit. Column **D** (D1–D5) represents the 24-month follow-up visit. Row 1 (A1, B1, C1, D1) shows the drusen volume maps with volume quantification in the 5-mm fovea-centered circle. Row 2 (A2, B2, C2, D2) shows the final outlines with HRF area measurements in the 5-mm circle centered on the fovea. Row 3 (A3, B3, C3, D3) shows the en face SS-OCTA sub-RPE slabs obtained using segmentation boundaries between 64 and 400 μm under Bruch’s membrane. Choroidal hypotransmission defects caused by HRF can be seen on en face sub-RPE slabs and corresponding color-framed B-scans (rows 4–5). At baseline (A1–A5), the eye showed a drusen volume of 0.09 mm^3^ (A1) with an HRF area of 0.07 mm^2^ (A2) within the 5-mm circle. After 12 months of follow-up (B1–B5), the drusen volume remained stable (B1), while the HRF area (B2) decreased. Two large hyperTDs emerged (yellow arrows, B3). By the 18-month follow-up, the HRF area had further decreased (C1 and C2), and the previously developed large hyperTDs had enlarged while a new one had formed (yellow arrows, C3). At the 24-month visit, both large hyperTDs showed continued growth (yellow arrows, D3), while drusen volume (D1) and HRF area (D2) decreased. HRF = hyperreflective foci; hyperTD = hypertransmission defect; iAMD = intermediate age-related macular degeneration; RPE = retinal pigment epithelium; SS-OCTA = swept-source OCT angiography.

**Figure 4:**
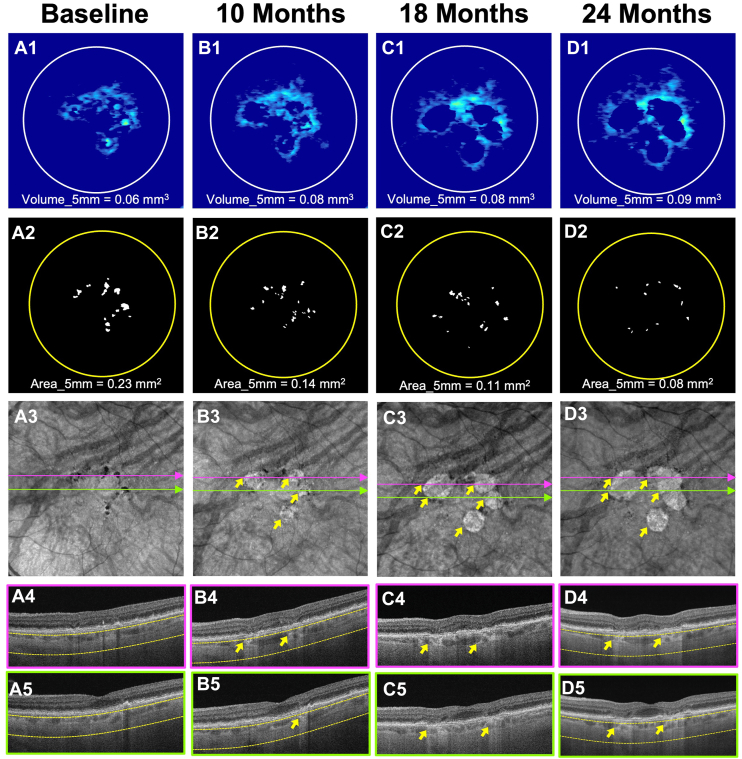
Longitudinal SS-OCTA imaging from an iAMD eye meeting the baseline HRF area (≥0.07 mm^2^) criterion for enrollment in the second clinical trial design. Column **A** (A1–A5) represents the baseline visit. Column **B** (B1–B5) represents the 10-month follow-up visit. Column **C** (C1–C5) represents the 18-month follow-up visit. Column **D** (D1–D5) represents the 24-month follow-up visit. Row 1 (A1, B1, C1, D1) shows the drusen volume maps with volume quantification in the 5-mm fovea-centered circle. Row 2 (A2, B2, C2, D2) shows the final outlines with HRF area measurements in the 5-mm circle centered on the fovea. Row 3 (A3, B3, C3, D3) shows the en face SS-OCTA sub-RPE slabs obtained using segmentation boundaries between 64 and 400 μm under Bruch’s membrane. Choroidal hypotransmission defects caused by HRF can be seen on en face sub-RPE slabs and corresponding color-framed B-scans (rows 4–5). At baseline (A1–A5), the eye showed a drusen volume of 0.06 mm^3^ (A1) with an HRF area of 0.23 mm^2^ (A2) within the 5-mm circle. After 10 months of follow-up (B1–B5), the drusen volume slightly increased (B1), while the HRF area (B2) decreased. Four large hyperTDs emerged (yellow arrows, B3). By the 18-month follow-up, drusen volume had remained stable while HRF area had further decreased (C1 and C2), and the previously developed large hyperTDs continued to enlarge (yellow arrows, C3). At the 24-month visit, both large hyperTDs showed continued growth and eventually merged together (yellow arrows, D3), while drusen volume (D1) and HRF area (D2) showed minimal changes. HRF = hyperreflective foci; hyperTD = hypertransmission defect; iAMD = intermediate age-related macular degeneration; RPE = retinal pigment epithelium; SS-OCTA = swept-source OCT angiography.

A total of 79 eyes from 62 patients met the inclusion criterion of having an area of HRF ≥0.07 mm^2^ either at baseline or during follow-up but prior to the onset of their first large hyperTD. Of these patients, 61% were women. The median follow-up time was 35.4 months (95% CI: 31.8–47.4 months). After 12 months, 14 eyes (17.7%) had developed a large hyperTD, increasing to 32 eyes (40.5%) by 24 months. Among the 79 eyes in this group, 53 (67.1%) developed ≥1 large hyperTD during follow-up. The median time to the onset of a large hyperTD was 17.9 months (95% CI: 14.7–24.2 months). The mean annual slope of the square-root-transformed large hyperTD area growth was slightly higher at 0.161 mm/year (SD = 0.172 at the eye level). The modeled 25% reduction in progression in this cohort corresponded to a mean treatment effect of 0.040 mm/year, while a 50% reduction equated to 0.080 mm/year (Table 1). Power calculations were conducted using these effect sizes in combination with assumed SDs of 0.16, 0.17, and 0.18. Figures 3 and 4 show 2 examples of eyes with baseline HRF area >0 mm^2^ that developed large hyperTDs within 2 years of enrollment.

### Comparison of Progression Slopes across Designs

Using the linear mixed-effects model described earlier, we estimated annual progression rates of square root large hyperTD area for each inclusion definition. Among eyes with DV ≥0.20 mm^3^ and any HRF, the mean slope was approximately 0.158 mm/year, with a between-subject SD of 0.180 mm/year. For eyes with HRF ≥0.07 mm^2^, the mean slope was approximately 0.167 mm/year, with an SD of 0.170 mm/year. These mixed-effects estimates represent population-averaged progression rates and incorporate subject-level variability through random intercepts and slopes.

For each inclusion definition, the distribution of per-eye annual slopes was right-skewed (Figure S5, available at https://www.ophthalmologyscience.org). In eyes with DV ≥0.20 mm^3^ and HRF >0 mm^2^, the LSR slopes had a 10th percentile (P10) = 0, median = 0.0906, and P90 = 0.4026, with 30 of 76 eyes (39%) showing no measurable lesion growth. The corresponding mixed-effects model yielded P10 = 0.0065, median = 0.0917, and P90 = 0.4200, with no zero slopes. In the HRF ≥0.07 mm^2^ subset, the LSR slopes had a P10 = 0, median = 0.1277, and P90 = 0.3682 (26/79 eyes with zero slopes), whereas the mixed-effects model produced P10 = 0.0065, median = 0.1227, and P90 = 0.3982, with no zero slopes observed. The absence of zero slopes in the mixed-effects model reflects its continuous Gaussian assumption and does not imply lesion development in eyes that remained lesion-free.

### Sample Size and Power Calculation

Sample size estimates yielding 80% power were modeled under 3 prespecified significance thresholds by using 2-sided alpha levels of 0.15, 0.10, and 0.05, based on the slope distributions obtained from our model.

For the DV ≥0.20 and HRF >0 trial design (Table 2) at a 2-sided alpha of 0.15, the detection of a 25% reduction in the annual rate of change in the square root of the total large hyperTD area required between 482 and 594 total eyes (241–297 per arm), depending on the assumed SD of 0.18 to 0.20. In contrast, a 50% reduction in progression could be detected with only 122 to 150 eyes (61–75 per arm), showing the large gain in efficiency when targeting a more pronounced treatment effect. At a 2-sided alpha of 0.10, the sample size requirements increased modestly. A 25% reduction required 572 to 706 eyes (286–353 per arm) while a 50% reduction was detectable with 144 to 178 eyes (72–89 per arm), depending on the assumed SD. Using a more stringent 2-sided alpha of 0.05, we found the required sample size for detecting a 25% reduction increased to a range from 726 to 896 eyes (363–448 per arm). For a 50% reduction, the required sample size ranged from 184 to 226 eyes (92–113 per arm) across SD values of 0.18 to 0.20. Additional sensitivity scenarios were evaluated using SDs increased by 10%, 20%, and 30% to reflect potential multicenter variability; the detailed results are presented in Table 2.

**Table 2:** Required Sample Sizes of Eyes with DV ≥0.20 mm^3^ and Any HRF Needed to Have an 80% Power to Detect a Slowing in the Square Root Total Annual Rate of Change in the Area of Large hyperTDs (mm)

Trial Design	2-Sided Alpha	SD	Number of Total Eyes in a 2-Arm Study Designed to Detect a Reduction in Annual Rate of Change in Both Active vs. Control Arms (mm/Year) [Minimum Difference in Mean Annual Slope Required to Achieve p<2-Sided Alpha]					
			0.0375 [0.0236], (25%)	0.0450 [0.0284], (30%)	0.0525 [0.0331], (35%)	0.0600 [0.0378], (40%)	0.0675 [0.0426], (45%)	0.0750 [0.0472], (50%)
DV ≥0.20 mm^3^; HRF area >0 mm^2^	0.15	0.18	482 (241:241) 536 (268:268)	334 (167:167) 372 (186:186)	246 (123:123) 274 (137:137)	190 (95:95) 212 (106:106)	150 (75:75) 168 (84:84)	122 (61:61) 136 (68:68)
		0.19	536 (268:268) 596 (298:298)	372 (186:186) 414 (207:207)	274 (137:137) 306 (153:153)	210 (105:105) 234 (117:117)	166 (83:83) 186 (93:93)	136 (68:68) 152 (76:76)
		0.20	594 (297:297) 660 (330:330)	414 (207:207) 460 (230:230)	304 (152:152) 338 (169:169)	234 (117:117) 260 (130:130)	184 (92:92) 206 (103:103)	150 (75:75) 168 (84:84)
		0.21	654 (327:327) 728 (364:364)	456 (228:228) 508 (254:254)	334 (167:167) 372 (186:186)	256 (128:128) 286 (143:143)	204 (102:102) 228 (114:114)	166 (83:83) 186 (93:93)
		0.23	784 (392:392) 872 (436:436)	546 (273:273) 608 (304:304)	402 (201:201) 448 (224:224)	308 (154:154) 344 (172:172)	244 (122:122) 272 (136:136)	198 (99:99) 220 (110:110)
		0.25	926 (463:463) 1030 (515:515)	644 (322:322) 716 (358:358)	474 (237:237) 528 (264:264)	364 (182:182) 406 (203:203)	288 (144:144) 320 (160:160)	234 (117:117) 260 (130:130)
	0.10	0.18	572 (286:286) 636 (318:318)	398 (199:199) 444 (222:222)	294 (147:147) 328 (164:164)	224 (112:112) 250 (125:125)	178 (89:89) 198 (99:99)	144 (72:72) 160 (80:80)
		0.19	638 (319:319) 710 (355:355)	444 (222:222) 494 (247:247)	326 (163:163) 364 (182:182)	250 (125:125) 278 (139:139)	198 (99:99) 220 (110:110)	162 (81:81) 180 (90:90)
		0.20	706 (353:353) 786 (393:393)	490 (245:245) 546 (273:273)	362 (181:181) 404 (202:202)	278 (139:139) 310 (155:155)	220 (110:110) 246 (123:123)	178 (89:89) 198 (99:99)
		0.21	778 (389:389) 866 (433:433)	540 (270:270) 600 (300:300)	398 (199:199) 444 (222:222)	306 (153:153) 340 (170:170)	242 (121:121) 270 (135:135)	196 (98:98) 218 (109:109)
		0.23	932 (466:466) 1036 (518:518)	648 (324:324) 720 (360:360)	476 (238:238) 530 (265:265)	366 (183:183) 408 (204:204)	290 (145:145) 324 (162:162)	234 (117:117) 260 (130:130)
		0.25	1102 (551:551) 1226 (613:613)	766 (383:383) 852 (426:426)	564 (282:282) 628 (314:314)	432 (216:216) 480 (240:240)	342 (171:171) 380 (190:190)	278 (139:139) 310 (155:155)
	0.05	0.18	726 (363:363) 808 (404:404)	506 (253:253) 564 (282:282)	372 (186:186) 414 (207:207)	286 (143:143) 318 (159:159)	226 (113:113) 252 (126:126)	184 (92:92) 206 (103:103)
		0.19	808 (404:404) 898 (449:449)	562 (281:281) 626 (313:313)	414 (207:207) 460 (230:230)	318 (159:159) 354 (177:177)	252 (126:126) 280 (140:140)	204 (102:102) 228 (114:114)
		0.20	896 (448:448) 996 (498:498)	624 (312:312) 694 (347:347)	458 (229:229) 510 (255:255)	352 (176:176) 392 (196:196)	278 (139:139) 310 (155:155)	226 (113:113) 252 (126:126)
		0.21	988 (494:494) 1098 (549:549)	686 (343:343) 764 (382:382)	506 (253:253) 564 (282:282)	388 (194:194) 432 (216:216)	306 (153:153) 340 (170:170)	250 (125:125) 278 (139:139)
		0.23	1184 (592:592) 1316 (658:658)	824 (412:412) 916 (458:458)	606 (303:303) 674 (337:337)	464 (232:232) 516 (258:258)	368 (184:184) 410 (205:205)	298 (149:149) 332 (166:166)
		0.25	1398 (699:699) 1554 (777:777)	972 (486:486) 1080 (540:540)	714 (357:357) 794 (397:397)	548 (274:274) 610 (305:305)	434 (217:217) 484 (242:242)	352 (176:176) 392 (196:196)

DV = drusen volume; HRF = hyperreflective foci; hyperTD = hypertransmission defect; SD = standard deviation.

The table presents sample size requirements under 3 significance thresholds (2-sided α = 0.15, 0.10, and 0.05) for a range of assumed SDs, including values increased by 10%, 20%, and 30% to reflect potential multicenter variability. In each cell, the first row indicates the sample size required under the specified assumptions, and the second row provides the corresponding value after applying a 10% adjustment. A 2-sided α = 0.15, 0.10, and 0.05 correspond, respectively, to 1-sided α = 0.075, 0.05, and 0.025.

For the HRF ≥0.07 trial design (Table 3), sample size estimates yielding 80% power were consistently lower across all assumptions due to the higher baseline progression rate and lower SDs observed in this group. A 2-sided alpha of 0.15, detecting a 25% reduction in slope, required between 334 and 424 eyes (167–212 per arm) for SDs of 0.16 to 0.18, while a 50% reduction required only 86 to 132 eyes (43–66 per arm). At a 2-sided alpha of 0.10, the required sample sizes increased slightly, with 398 to 504 eyes (199–252 per arm) needed to detect a 25% reduction, and 102 to 128 eyes (51–64 per arm) for a 50% reduction. Finally, at a 2-sided alpha of 0.05, a 25% reduction required between 506 and 638 eyes (253–319 per arm), while a 50% reduction could be detected with 128 to 162 eyes (64–81 per arm), depending on the assumed SD. Power calculations incorporating increased SDs are reported in Table 3.

**Table 3:** Required Sample Sizes of Eyes with HRF Area ≥0.07 mm^2^ to Have an 80% Power to Detect a Slowing in the Square Root Total Annual Rate of Change in the Area of Large hyperTDs (mm)

Trial Design	2-Sided Alpha	SD	Number of Total Eyes in a 2-Arm Study Designed to Detect a Reduction in Annual Rate of Change in Both Active vs. Control Arms (mm/Year) [Minimum Difference in Mean Annual Slope Required to Achieve p<2-Sided Alpha]					
			0.0400 [0.0280], (25%)	0.0480 [0.0336], (30%)	0.0560 [0.0391], (35%)	0.0640 [0.0447], (40%)	0.0720 [0.0503], (45%)	0.0800 [0.0560], (50%)
HRF area ≥0.07 mm^2^	0.15	0.16	334 (167:167) 372 (186:186)	234 (117:117) 260 (130:130)	172 (86:86) 192 (96:96)	132 (66:66) 148 (74:74)	104 (52:52) 116 (58:58)	86 (43:43) 96 (48:48)
		0.17	378 (189:189) 420 (210:210)	264 (132:132) 294 (147:147)	194 (97:97) 216 (108:108)	148 (74:74) 166 (83:83)	118 (59:59) 132 (66:66)	96 (48:48) 108 (54:54)
		0.18	424 (212:212) 472 (236:236)	294 (147:147) 328 (164:164)	218 (109:109) 244 (122:122)	166 (83:83) 186 (93:93)	132 (66:66) 148 (74:74)	108 (54:54) 120 (60:60)
		0.19	472 (236:236) 526 (263:263)	328 (164:164) 366 (183:183)	242 (121:121) 270 (135:135)	186 (93:93) 208 (104:104)	146 (73:73) 164 (82:82)	120 (60:60) 134 (67:67)
		0.205	548 (274:274) 610 (305:305)	382 (191:191) 426 (213:213)	280 (140:140) 312 (156:156)	216 (108:108) 240 (120:120)	170 (85:85) 190 (95:95)	138 (69:69) 154 (77:77)
		0.22	632 (316:316) 704 (352:352)	440 (220:220) 490 (245:245)	324 (162:162) 360 (180:180)	248 (124:124) 276 (138:138)	196 (98:98) 218 (109:109)	160 (80:80) 178 (89:89)
	0.10	0.16	398 (199:199) 444 (222:222)	278 (139:139) 310 (155:155)	204 (102:102) 228 (114:114)	156 (78:78) 174 (87:87)	124 (62:62) 138 (69:69)	102 (51:51) 114 (57:57)
		0.17	450 (225:225) 500 (250:250)	312 (156:156) 348 (174:174)	230 (115:115) 256 (128:128)	176 (88:88) 196 (98:98)	140 (70:70) 156 (78:78)	114 (57:57) 128 (64:64)
		0.18	504 (252:252) 560 (280:280)	350 (175:175) 390 (195:195)	258 (129:129) 288 (144:144)	198 (99:99) 220 (110:110)	156 (78:78) 174 (87:87)	128 (64:64) 144 (72:72)
		0.19	560 (280:280) 624 (312:312)	390 (195:195) 434 (217:217)	288 (144:144) 320 (160:160)	220 (110:110) 246 (123:123)	174 (87:87) 194 (97:97)	142 (71:71) 158 (79:79)
		0.205	652 (326:326) 726 (363:363)	454 (227:227) 506 (253:253)	334 (167:167) 372 (186:186)	256 (128:128) 286 (143:143)	202 (101:101) 226 (113:113)	164 (82:82) 184 (92:92)
		0.22	750 (375:375) 834 (417:417)	522 (261:261) 580 (290:290)	384 (192:192) 428 (214:214)	294 (147:147) 328 (164:164)	234 (117:117) 260 (130:130)	190 (95:95) 212 (106:106)
	0.05	0.16	506 (253:253) 564 (282:282)	352 (176:176) 392 (196:196)	260 (130:130) 290 (145:145)	200 (100:100) 224 (112:112)	158 (79:79) 176 (88:88)	128 (64:64) 144 (72:72)
		0.17	570 (285:285) 634 (317:317)	396 (198:198) 440 (220:220)	292 (146:146) 326 (163:163)	224 (112:112) 250 (125:125)	178 (89:89) 198 (99:99)	144 (72:72) 160 (80:80)
		0.18	638 (319:319) 710 (355:355)	444 (222:222) 494 (247:247)	328 (164:164) 366 (183:183)	252 (126:126) 280 (140:140)	200 (100:100) 224 (112:112)	162 (81:81) 180 (90:90)
		0.19	712 (356:356) 792 (396:396)	494 (247:247) 550 (275:275)	364 (182:182) 406 (203:203)	280 (140:140) 312 (156:156)	222 (111:111) 248 (124:124)	180 (90:90) 200 (100:100)
		0.205	828 (414:414) 920 (460:460)	576 (288:288) 640 (320:320)	424 (212:212) 472 (236:236)	326 (163:163) 364 (182:182)	258 (129:129) 288 (144:144)	210 (105:105) 234 (117:117)
		0.22	952 (476:476) 1058 (529:529)	662 (331:331) 736 (368:368)	488 (244:244) 544 (272:272)	374 (187:187) 416 (208:208)	296 (148:148) 330 (165:165)	240 (120:120) 268 (134:134)

HRF = hyperreflective foci; hyperTD = hypertransmission defect; SD = standard deviation.

The table presents sample size requirements under 3 significance thresholds (2-sided α = 0.15, 0.10, and 0.05) for a range of assumed SDs, including values increased by 10%, 20%, and 30% to reflect potential multicenter variability. In each cell, the first row indicates the sample size required under the specified assumptions, and the second row provides the corresponding value after applying a 10% adjustment. A 2-sided α = 0.15, 0.10, and 0.05 correspond, respectively, to 1-sided α = 0.075, 0.05, and 0.025.

## Discussion

By using the growth of large choroidal hyperTDs as a structural endpoint, we proposed and modeled 2 clinical trial designs aimed at evaluating therapies to slow the progression of iAMD to early atrophy. Our results show that both DV and HRF are effective biomarkers for identifying high-risk iAMD eyes, and that the square root–transformed slope of growth for the total area of large hyperTDs over 2 years can serve as a sensitive, continuous outcome measurement. We use our natural history study to produce simulations to estimate the power of these clinical trials for various expected treatment effects.

The selection of the growth rate of large hyperTDs as the primary efficacy endpoint is consistent with the recent consensus reached by leading AMD experts at the 2024 Ryan Initiative for Macular Research meeting, as detailed by Guymer et al.^28^ In line with current regulatory guidance, the US Food and Drug Administration has emphasized the importance of continuous, quantitative outcome measures for early-stage disease, favoring endpoints that capture subtle but clinically meaningful changes over time.^43^ Unlike traditional event-based endpoints, such as the onset of atrophy, the slope of large hyperTD area growth integrates both lesion onset and subsequent enlargement into a single continuous progression metric, increasing sensitivity to treatment effects over relatively short follow-up periods and enabling efficient phase II trial designs.^13^ This clinical trial framework enables the evaluation of therapeutic effects during the transition from iAMD to early GA using a single, continuous structural endpoint. Importantly, this does not imply biological or regulatory equivalence between iAMD and established GA, nor does it replace the need for stage-specific analyses or confirmatory trials in eyes with more advanced atrophy. Within this framework, the duration of follow-up can be adapted to the study objective: longer follow-up increases statistical power and cost efficiency by allowing the same slope-based endpoint to capture both delayed lesion onset and subsequent growth. Data generated from such iAMD trials can therefore inform the design assumptions, effect-size expectations, and regulatory strategy of subsequent trials in eyes with established GA. The Ryan Initiative for Macular Research panel specifically endorsed en face OCT-based continuous structural endpoints for iAMD trials, highlighting the appearance and growth of large hyperTDs and ellipsoid zone (EZ) loss as key candidate biomarkers.^28^

Large hyperTDs are a robust and easily quantifiable structural biomarker of disease progression in iAMD.^32^ Detection on en face OCT is highly reproducible after standardized training and is not platform-dependent, supporting its use as a scalable structural trial endpoint.^32^^,^^44^ Once hyperTDs reach a GLD ≥250 μm, they have been shown to persist in 99.6% of cases over time,^45^ and to correspond to complete RPE and outer retinal atrophy when assessed comprehensively.^7^^,^^44^ Although large hyperTDs may precede classic GA as defined by color fundus photography or fundus autofluorescence, longitudinal studies consistently demonstrate their progression to conventional GA over time.^13^^,^^26^^,^^27^^,^^44^^,^^46^^,^^47^ Their reliable detectability on en face sub-RPE slabs and confirmability on corresponding B-scans support their suitability as longitudinal trial endpoints.

In contrast, EZ loss has important limitations as a primary endpoint in iAMD trials. Ellipsoid zone reflectivity and segmentation are highly sensitive to technical factors, signal attenuation over drusen, and anatomical variability, complicating automated measurement and longitudinal reproducibility. Moreover, apparent EZ disruptions may reflect transient optical effects rather than irreversible degeneration.^48^, ^49^, ^50^, ^51^, ^52^, ^53^, ^54^, ^55^, ^56^, ^57^ While associations between EZ-related metrics and functional outcomes have been reported, these approaches remain instrument-dependent and operationally complex. By comparison, large hyperTDs provide a more robust, scalable, and biologically grounded marker of progression for early-phase iAMD trials.

Based on these observations, we decided to use the growth of large hyperTDs as the primary structural endpoint for the proposed designs. Eligible eyes are required to have no large hyperTDs at baseline, allowing both lesion onset and subsequent enlargement to be captured over time.^14^ Ideally, to be feasible and efficient, a practical trial design must detect treatment effects within a 2-year time frame. This necessitates the selection of eyes at very high risk of progression to ensure that a sufficient proportion of participants develop measurable endpoints during the study. The use of a single 6 × 6 mm OCT raster enables simultaneous assessment of large hyperTDs, DV, and HRF area, streamlining screening and longitudinal follow-up while ensuring consistent endpoint evaluation across visits.^14^^,^^15^^,^^26^^,^^27^^,^^29^^,^^34^

Drusen volume is a well-established and widely validated OCT biomarker, with automated segmentation now incorporated into many commercial spectral-domain OCT and SS-OCT platforms.^11^, ^12^, ^13^^,^^29^^,^^38^ However, a subset of iAMD eyes progresses despite a limited drusen burden, for which HRF area provides complementary risk stratification.^14^^,^^15^ By combining these biomarkers, the proposed designs capture a larger proportion of eyes at high risk of progression while balancing maximal enrichment with operational feasibility. Accordingly, one design relies on the presence of HRF without area quantification, whereas the second design leverages HRF area thresholds to achieve maximal enrichment.

In the first trial design (DV ≥0.20 mm^3^ and any detectable HRF), our natural history study estimates a mean annual slope of square root of large hyperTD area of 0.153 mm/year (SD = 0.188), with 40% of eyes contributing zero slopes due to the absence of lesion formation over the 2-year follow-up. Despite this heterogeneity, slope-based modeling demonstrated that even modest treatment effects, such as a 25% reduction in progression, could be detected with feasible sample sizes, depending on the statistical thresholds applied (Table 2). Under a 2-sided alpha level of 0.10 and a modeled 50% treatment effect, 72–89 subjects (1 eye per subject) per arm would provide 80% power (Table 2), representing a practical compromise between statistical power and enrollment feasibility. This trial design has been recently endorsed by the US Food and Drug Administration, which approved the use of large hyperTD growth as a primary efficacy endpoint in a phase IIb study of oral tonabersat, a connexin43 hemichannel inhibitor, for iAMD. This tonabersat study will enroll patients with a central DV ≥0.20 mm^3^, HRF area >0 mm^2^, and no large hyperTDs at baseline. The primary endpoint is the growth rate of large hyperTDs over 2 years, with secondary endpoints including their incidence at 1 and 2 years. Notably, the 1-year incidence will help guide future phase III trial planning.

In the second trial design, which enrolled eyes with HRF area ≥0.07 mm^2^ regardless of DV, the mean annual slope of large hyperTD square root area was slightly higher at 0.161 mm/year, with a somewhat lower SD (SD = 0.172). This combination of faster progression and reduced variability translated into improved statistical efficiency. As shown in Table 3, a 50% reduction in hyperTD growth could be detected with 80% power with just 51 to 64 subjects per arm, assuming a 2-sided alpha of 0.10. This increases the attractiveness of HRF-based strategies when optimizing a trial design and minimizing sample size. However, widespread implementation may be constrained by the current lack of available tools for fully automated HRF quantification. At present, HRF area measurement still requires semiautomated processing with manual input, which could limit scalability in larger multicenter trials. Nonetheless, these results highlight the value of HRF as a potent risk stratifier and support its continued development as a trial inclusion criterion.

Although these trial models were developed using SS-OCTA, multiple cross-platform studies have demonstrated strong concordance between SS-OCT and spectral-domain OCT for DV, HRF area, and large hyperTD detection when identical en face slab definitions and algorithms are applied.^36^, ^37^, ^38^ Accordingly, the proposed designs are not device-specific, provided that imaging acquisition is standardized and the same device is used consistently for longitudinal follow-up within a trial. In multicenter trials, this is typically achieved through protocolized acquisition and centralized reading-center grading with certified graders and adjudication, which is the operational framework assumed by this design.

While enriching clinical trials with eyes at high risk of progression is essential to detect treatment effects within a 2-year timeframe, there is an inherent risk in enrolling eyes with lesions that are already too advanced to respond to therapy. Calcified drusen (CaD) exemplify this challenge: although strongly associated with progression to large hyperTDs,^19^ CaD likely represent a more advanced and less modifiable disease stage. Including such eyes indiscriminately may reduce sensitivity to treatment effects. Trial designs may therefore benefit from stratification by CaD presence or exclusion of CaD-positive eyes in early-phase studies to preserve a biologically responsive population. These considerations also reinforce the need to move beyond outdated classification systems, such as the Beckman classification, which relies on color fundus photography and clinical examination and does not incorporate modern OCT-derived biomarkers.^2^ Grouping all iAMD eyes into a single category, without accounting for critical features such as DV, HRF, calcified drusen, or large hyperTDs, dilutes prognostic accuracy and complicates trial design. A modern classification system that integrates OCT imaging and stratifies iAMD eyes by true biological risk is essential, not only to improve patient care but also to support rational, efficient, and targeted clinical research.

While functional outcome measures remain important for assessing patient-relevant benefit and safety, structural endpoints are better suited to detect early biological effects over short study durations. In this context, the growth rate of large hyperTDs provides a biologically proximal measure that integrates lesion onset and enlargement. Functional measures should be incorporated as secondary or exploratory outcomes, while ongoing and future studies prospectively define structure-function relationships using longitudinal multimodal data. This includes correlating longitudinal changes in the appearance and growth of large hyperTDs with functional measures, including best-corrected visual acuity, microperimetry, contrast sensitivity, and reading performance. Mixed-effects or joint models can be used to assess whether changes in structural progression predict concurrent or subsequent functional decline.

Several limitations must be acknowledged. First, this work is based on a single-center, longitudinal natural history cohort, which may not fully capture disease heterogeneity or imaging variability across different clinical settings. In addition, some of the analytical tools used in this study are currently available primarily in specialized research environments and centralized reading centers, and measurements of large hyperTDs and HRF may vary across centers and algorithms. We attempted to account for this by modeling trial outcomes under a range of plausible SD assumptions. Because the analyses rely on observational natural history data with irregular follow-up, no imputation was performed, and progression slopes were estimated from observed data only; handling of missing data in interventional trials will be prespecified in the trial protocol and statistical analysis plan. Moreover, while square root transformation of hyperTD area is standard in GA modeling, its role as a surrogate for visual function in iAMD remains to be established, warranting future structure–function correlation studies. Finally, because HRF area quantification is currently more resource-intensive than DV and hyperTD measurement, we intentionally present a complementary trial design that does not require HRF area quantification, offering a scalable alternative when operational considerations outweigh maximal enrichment.

Despite these limitations, our results demonstrate the flexibility and efficiency of slope-based structural endpoints for accelerating drug development in iAMD. By targeting high-risk eyes using OCT biomarkers, clinical trials can be designed to detect biological treatment effects over shorter durations and with fewer participants. These findings support a broader shift toward imaging-based enrichment and progression metrics in early AMD, a shift analogous to biomarker-driven approaches in both Alzheimer disease and oncological studies.

In conclusion, we propose 2 practical clinical trial designs for iAMD that use OCT-derived biomarkers and the growth rate of large hyperTDs as a continuous structural endpoint. These designs offer a promising framework for evaluating treatments that may delay the onset of atrophy, slow its progression, and preserve macular function in patients with intermediate AMD.

## Declaration of Generative AI and AI-Assisted Technologies in the Writing Process

During the preparation of this work, the authors used ChatGPT5.2 to improve the manuscript’s language and readability. After using this tool, the authors reviewed and edited the content as needed and take full responsibility for the content of the publication.
