# Supplementary material for: Novel Clinical Trial Designs for Intermediate Age-Related Macular Degeneration
Source: Ophthalmol Sci. 2026 Mar 30;6(6):101179. doi: 10.1016/j.xops.2026.101179 (PMC13213873; doi:10.1016/j.xops.2026.101179)
Supplement: Figure S5 [file mmc1.pdf]

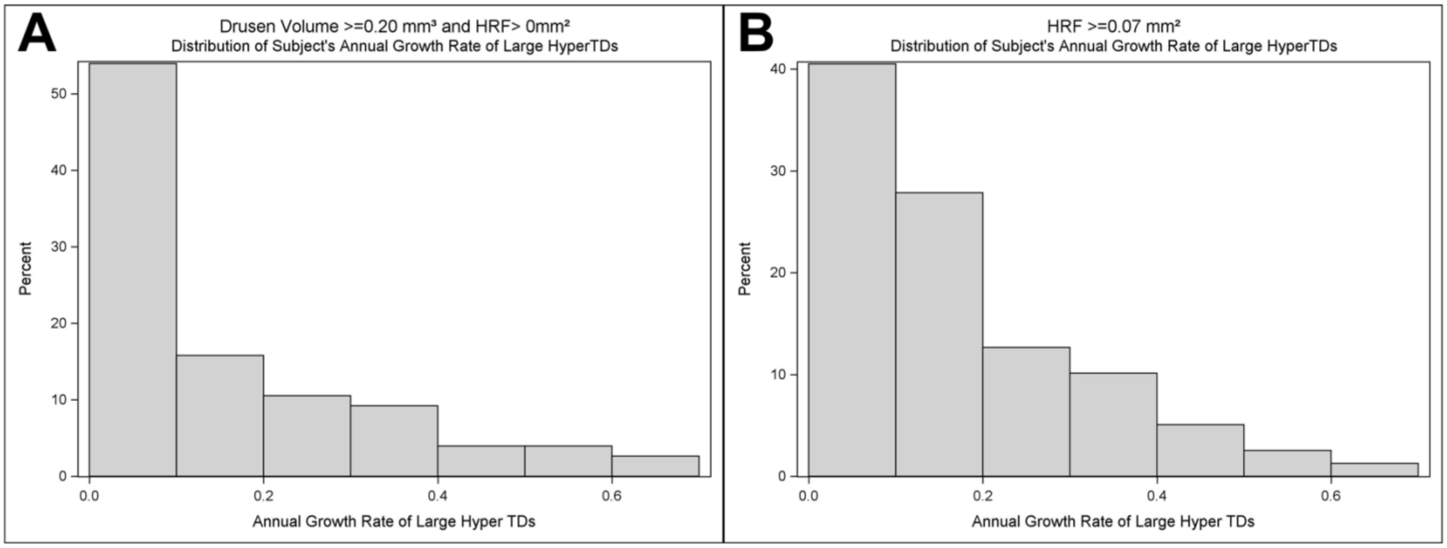

Figure S5. Distribution of per-eye annual growth rates of large hypertransmission defects (hyperTDs) in intermediate AMD eyes under each proposed clinical trial inclusion criterion. (A) Eyes with drusen volume ≥ 0.20 mm³ and any hyperreflective foci (HRF > 0 mm²). (B) Eyes with hyperreflective foci (HRF) ≥ 0.07 mm², regardless of drusen volume. Each histogram shows the frequency distribution (as a percentage of subjects) of the least-squares regression slopes of the square-root total hyperTD area (mm/year). The peak at zero represents eyes that did not develop large hyperTDs during follow-up, while the right-skewed tail reflects variable progression rates among eyes that did.
